# Experimental analysis of low-dip reverse fault dislocation effects on tunnel site models with different soil properties

**DOI:** 10.1038/s41598-024-51830-0

**Published:** 2024-01-13

**Authors:** Jianyi Zhang, Yijie Song, Zhongheng Li, Shihang Qu, Shuai Wang

**Affiliations:** 1 College of Geological Engineering, Institute of Disaster Prevention, Sanhe, 065201 China; 2 Hebei Key Laboratory of Earthquake Disaster Prevention and Risk Assessment, Sanhe, 065201 China; 3 Key Laboratory of Building Collapse Mechanism and Disaster Prevention, China Earthquake Administration, Sanhe, 065201 China; 4 Beijing Zhongkan Earthquake Prevention Technology Development Co., Ltd, Beijing, 100000 China

**Keywords:** Civil engineering, Solid Earth sciences, Engineering

## Abstract

The fortification system of the tunnel structure spanning the active fault, such as the failure mechanism and fault-resistant design (measures), has not been thoroughly established. In this study, the self-developed cross-fault large-scale bedrock dislocation loading device platform is utilized to carry out the model test of the tunnel structure and soil site of sand and cohesive soil when the low-angle reverse fault dislocation occurs, based on the earthquake damage. The results demonstrate that: (1) When the fault is staggered, the segmented flexible joint tunnel segment is more favorable in the cohesive soil site. (2) When compared to the cohesive soil tunnel structure site, the strain change of the tunnel structure in the sandy soil site is greater, with the vault increasing by roughly two times and the arch bottom increasing by nearly six times. After the tunnel is buried, the uplift range of the sand cover layer grows, revealing uneven deformation, and the rupture zone migrates to the footwall; hence, the sand site plays a “add seismic” role in the cross-fault tunnel structure. (3) Knowing the location and shape of the rupture range of the overburden soil caused by bedrock dislocation under different inclination angles and soil properties is required in the design in order to place the buried depth and segment length of the tunnel reasonably and take fault-resistant measures.

## Introduction

The surface faulting rupture site will rupture under the appropriate bedrock dislocation and damage the tunnel structure on it. With the building and operation of the cross-tunnel structure in an active fault-intensive area, tunnel designs that avoid or only conceptually cross the active fault have been unable to match the rapid development of tunnel engineering transportation. As a result, it is critical to investigate the site rupture mechanism of a cross-fault tunnel, as well as the failure characteristics of the tunnel construction and its anti-rupture measures. The location of the upper breakpoint of the overburden soil and how to destroy the tunnel structure at the location of the earthquake rupture dislocation have become important practical issues of concern to geological engineering, geotechnical engineering, and tunnel engineering researchers.

Currently, the research approaches for studying the failure of cross-fault tunnel structures and their failure mechanisms on site include analyzing earthquake damage cases^1–4^, conducting model tests^5–12^, and doing numerical simulations^13,15^. He et al. conducted an analysis of the data on damage to surrounding rock tunnels in fault rupture zones during the Wenchuan earthquake "5.12" and other significant earthquakes^1^. Their findings indicate that tunnels passing through fault rupture zones are prone to damage during earthquakes. The failure of tunnels in fault zones is primarily caused by the disparity in displacement between the surrounding rock in the fault zone and the more stable portion of the surrounding rock. Zhu et al.^2^ conducted a comprehensive review of the research conducted by the Tunnel Fault Disaster Mechanism Research Institute. They examined various models, including the engineering geological model, the mechanical model, and the dislocation displacement model. They also analyzed different methods, such as analytical methods, numerical simulation, and physical simulation. Additionally, the existing fault dislocation prevention and control measures, as well as the design and defense principles underlying them, are summarized. Through induction and classification, Li et al.^5^ compared and analyzed the key technologies in the current tunnel engineering physical simulation test system and proposed a test system classification method based on the size of the model. Guo et al.^6^ performed a 1:80 scale test using a dislocation model test instrument they designed themselves, together with digital image correlation technology. The objective was to investigate the process of shear rupture in the overburden resulting from the dislocation of a 60° dip-reverse fault. Shen^7^ used the design method of flexible joint segmented lining to conduct a shaking table test of a cross-fault tunnel in a hilly environment with a geological similarity ratio of 1:30. It is confirmed that it is applied to the structural design of cross-fault tunnels by improving the deformation adaptability of the tunnel structure. Sabagh et al.^8^ conducted a tunnel model test with a similarity ratio of 1:60 on a centrifuge for a reverse fault sand overburden site with a bedrock dislocation angle of 60°. The damage state of the tunnel was evaluated by the gradual increase in permanent ground displacement (PGD). Large-scale box test based on cross-fault tunnel seismic damage. Researchers can simulate tunnel stress under seismic fault dislocation and vibration conditions, investigate tunnel failure characteristics and causes, and devise effective fault prevention and control strategies.

Model test analysis and equipment for simulating cross-fault tunnels have garnered attention because of limited on-site seismic damage data, restrictions in research techniques, and funding. However, the test methods are mostly based on the shaking table model test, which simulates the input of near-fault ground motion. However, the shaking table model test cannot simulate the site rock and soil mass rupture caused by the sudden bedrock dislocation of the straight-down fault and the large deformation of the soil near the tunnel foundation or the dislocation of the surrounding rock, and the large deformation of the rock and soil around the tunnel is the main cause of the earthquake damage to the cross-fault tunnel. It is difficult to reasonably simulate the actual cross-fault tunnel structure and its overburden site by using the constant gravity dislocation loading device due to load reasons or the centrifuge model due to the small scale of the test due to the size of the hanging basket box.

As a result, we urgently need It is critical to develop a large-scale constant gravity model test device to simulate the dislocation loading mode of seism genic fault bedrock, analyze the response of tunnels across dip-slip faults under different site soil types, investigate the rupture distribution of overburden soil under dip-slip faults and the failure characteristics of internal tunnel structures, and provide basic data and a foundation for reasonably determining the damage and failure mechanisms of tunnels^16^.

So, this paper combines the large-scale test device platform of the cross-fault tunnel model developed by the research group to conduct research on the failure mechanism of the cross-fault segmented tunnel structure, as well as its site failure characteristics and anti-rupture measures, providing a certain reference value for the design and operation of the cross-fault tunnel structure.

## Design of a large-scale box test apparatus and scheme for cross-fault rupture tunnel models

### Large-scale model box test apparatus and instrumentation arrangement

The device adopts a self-developed large-scale experimental box platform, including a fault dislocation model platform (soil box, connecting device, base, actuator, and angle support); a bedrock fault dislocation input is simulated using an oil pressure loading device.

The test chamber measures 4.96 m long, 1.85 m wide, 1.85 m high, and 1.4 m high. On the front and back of the chamber, a high-strength organic transparent glass with a thickness of 0.025 m is fitted, and three vertical rigid ribs are positioned on the exterior side. The chamber's side is made of 0.015-m-high-strength steel plate. The box's bottom is made up of two totally rigid steel plates, an L-shaped movable steel plate and a fixed steel plate, which are used to model the dislocation of the top and lower plates of bedrock beneath the overburden dirt.

**Figure 1:**
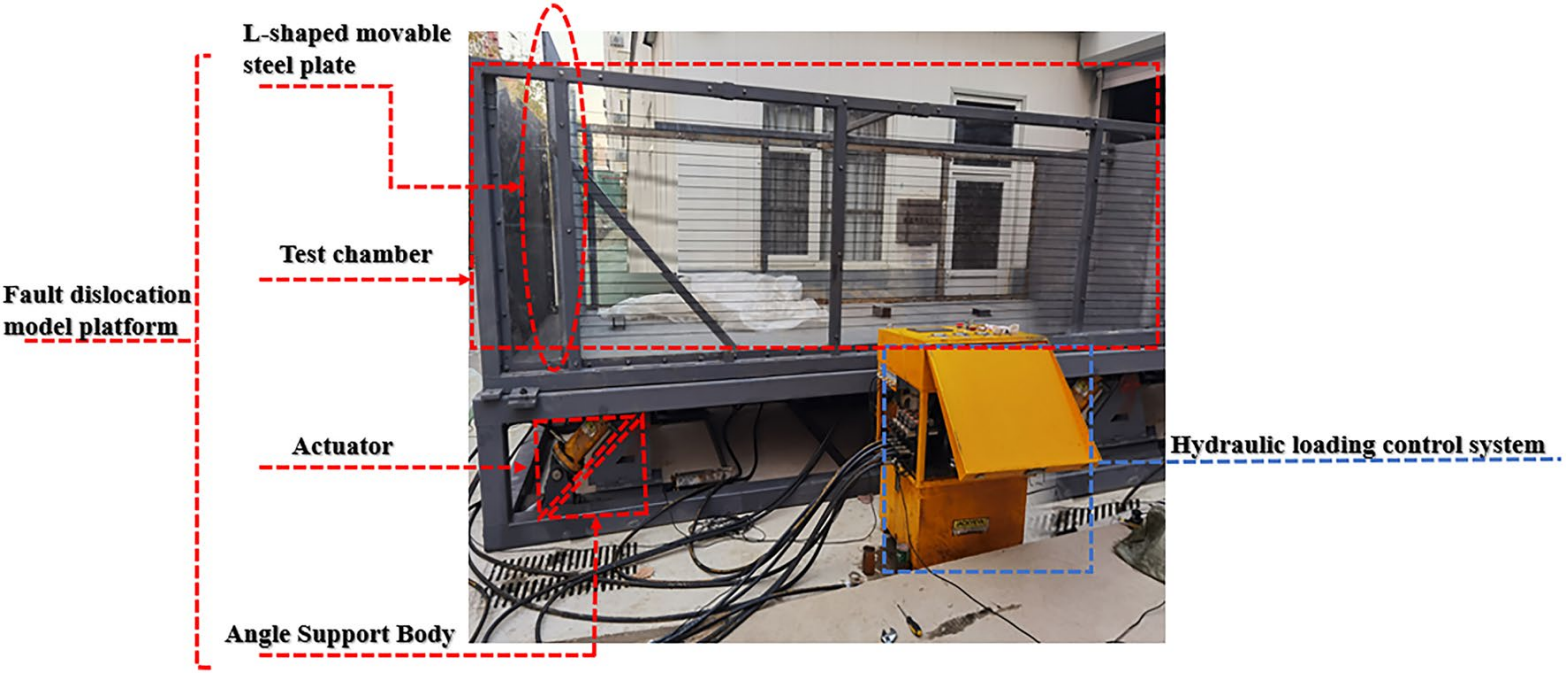
The actual platform of a model test apparatus for cross-fault tunnels.

**Figure 2:**
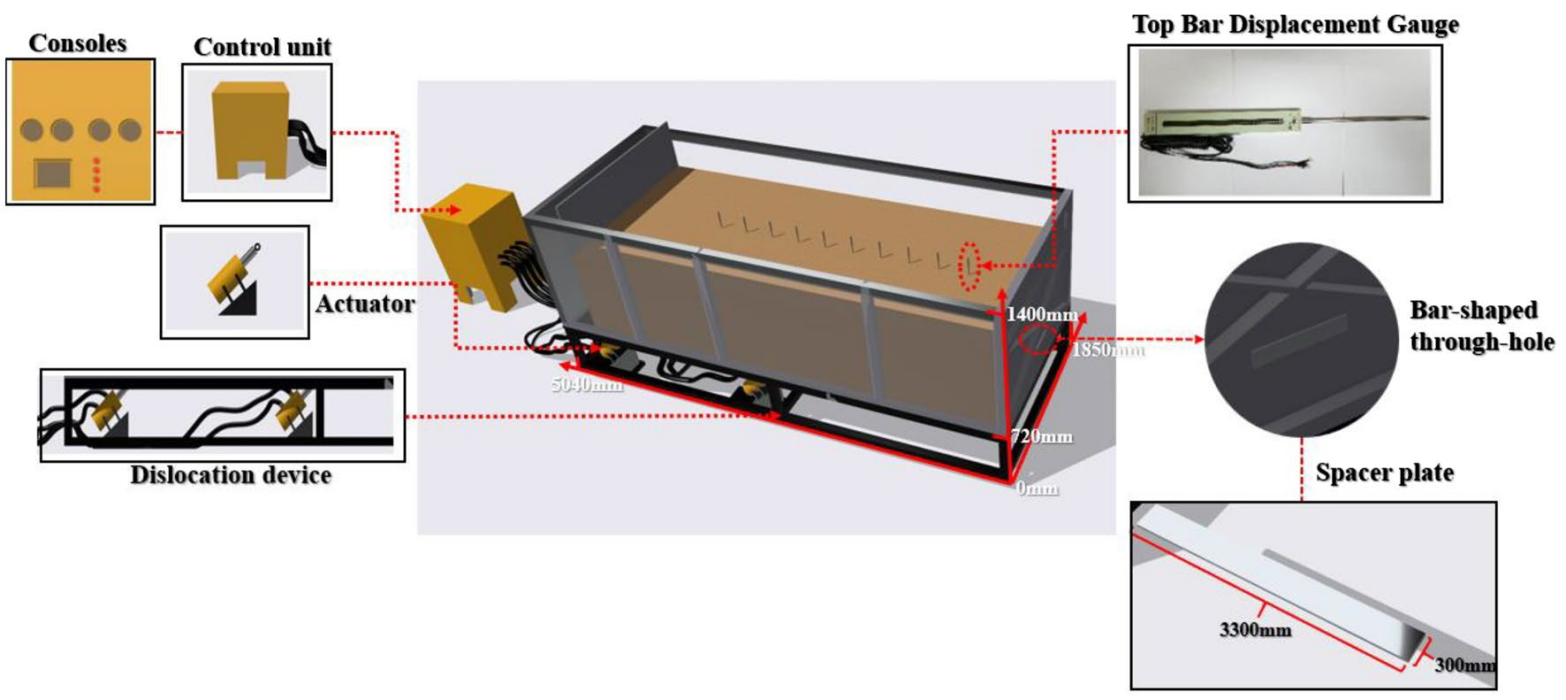
Three-dimensional diagram of the platform for the cross-fault tunnel model test apparatus.

All the steel plates and the box are connected by a soft canvas around them, and the loading device is placed on the L-shaped movable steel plate. The right side of the soil box is provided with a strip-through hole for inserting the transverse partition board. The partition board is used to bear the gravity caused by the soil compaction in the upper part of the model to avoid damage to the tunnel model. After the soil is rammed, the partition board is drawn out to better simulate the surrounding rock condition. The loading device adjusts the loading direction through the diagonal brace support on the base to simulate the tunnel structure failure under the fault dislocation at different tilt angles (Figs. 1, 2).

The advantage of this test device is that different test schemes can be formulated based on the tunnel's buried depth. By controlling the loading direction of the loading device, the failure of the tunnel structure under different dip angles and different fault dislocations of the normal and reverse faults is simulated in order to study the tunnel structure's failure mechanism under various working conditions.

### Pilot program design

The investigation primarily conducted four iterations of low-dip reverse fault dislocation simulation tests (Table 1), including two in a free field and two in a field with a tunnel structure. In this experiment, the tunnel model in the field test of the tunnel structure was constructed by assembling four sections of models with the same standard. The construction process followed the conventional procedures typically used for building tunnels.

**Table 1:** Model test working condition parameter table.

	Test number	Soil cover thickness (mm)	Soil type	Individual load (mm)	Total loading (mm)
Free-field	①	1000	Clays	10	110
	②	1000	Sandy soil	10	110
Sites with tunnels	③	1000	Clays	10	110
	④	1000	Sandy soil	10	110

The steps of each trial can be summarized as follows:

Complete the overall installation of the model box, adjust the actuator angle to 45°, and fix it. After the inspection is completed, lay a thin film at the joint between the base plate and the box to prevent soil leakage during the backfilling process. Once everything is in place, start the backfilling process.

Layer the soil and compact it. Fill 15 cm of soil each time and compact it to 10 cm. While compacting, bury sensors such as soil pressure gauges and accelerometers in the soil according to the layout of the testing instruments.

After compacting to a depth of 100 cm (measured from the surface of the soil), install a top bar displacement meter on the surface of the soil, and place cameras and other equipment above and in front of the soil box.

During the tunnel site testing, when compacting to a depth of 70 cm, according to the testing plan for the tunnel site, dig a hole in the soil with a depth of 40 cm and dimensions of 330 × 30 cm. Install strain gauges, displacement meters, and other sensors in the soil to set up the tunnel model. Lift the tunnel model and place it in the dug hole, connect the sections together, and secure them. Install partition boards and backfill the soil, compacting it to a depth of 100 cm.

Control the actuator to jack 10 mm for each sub-stage to record the data once. Each test is conducted under 11 different working conditions. In each working condition, ensure that the data acquisition instrument collects data according to the requirements of the sensor, and make sure to film the test using a camera.

### Model-making

Carry out a 1:30 similarity ratio modeling based on the developed multifunctional fault dislocation loading test device platform (Table 2).

**Table 2:** Soil parameters and their similarity constants in the model.

Physical quantity	*L:* Length	*ρ:* Density	*g:* Gravity acceleration	*τ:* Soil pressure
The constant of similarity of soil	${C}_{L}=30$	${C}_{\rho }=1$	${C}_{g}=1$	${C}_{\uptau }={C}_{L}\cdot {C}_{\rho }\cdot {C}_{g}=30$
The similarity constant of a tunnel	${C}_{L}=30$	${C}_{\rho }=1.25$	${C}_{g}=1$	–

*DL:* Rod displacement	*σ:* Stress	*ε:* Strain	*a:* Tunnel acceleration	*t:* Acceleration time
${D}_{L}=30$-	–	–	–	–
	${C}_{\sigma }={C}_{L}\cdot {C}_{\rho }\cdot {C}_{g}=37.5$	${C}_{\varepsilon }=1$	${C}_{a}=1$	${C}_{t}=\sqrt{{C}_{L}/{C}_{g}}=5.48$

Two types of soil, clay and sand, were used for the tests. Determining the similarity of the soils in the scaled-down modeling tests was challenging. Therefore, typical natural cohesive and sandy soils obtained from the field were used as soil samples.

**Figure 3:**
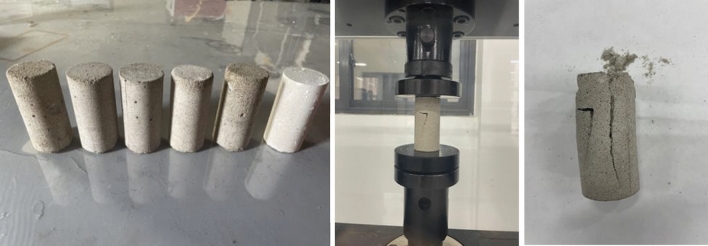
Preparation model similar material diagram.

**Figure 4:**
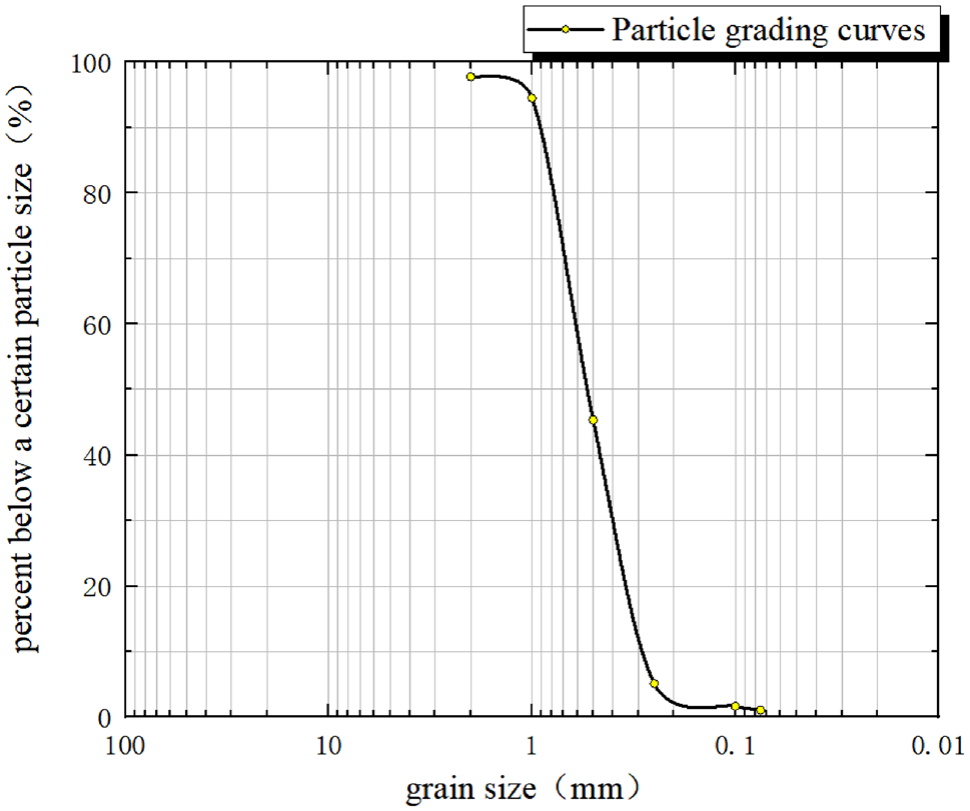
Particle grading curves for sandy soils.

The tunnel structural modeling materials include gypsum, cement, coarse sand, and fine sand. After conducting several proportioning tests based on a 1:30 similarity ratio, the appropriate ratio of these materials for the tunnel structure can be selected (Fig. 3). The coefficient of inhomogeneity, Cu, of the sandy soil samples was determined to be 2 through sieve testing and the particle grading curve (Fig. 4).

**Figure 5:**
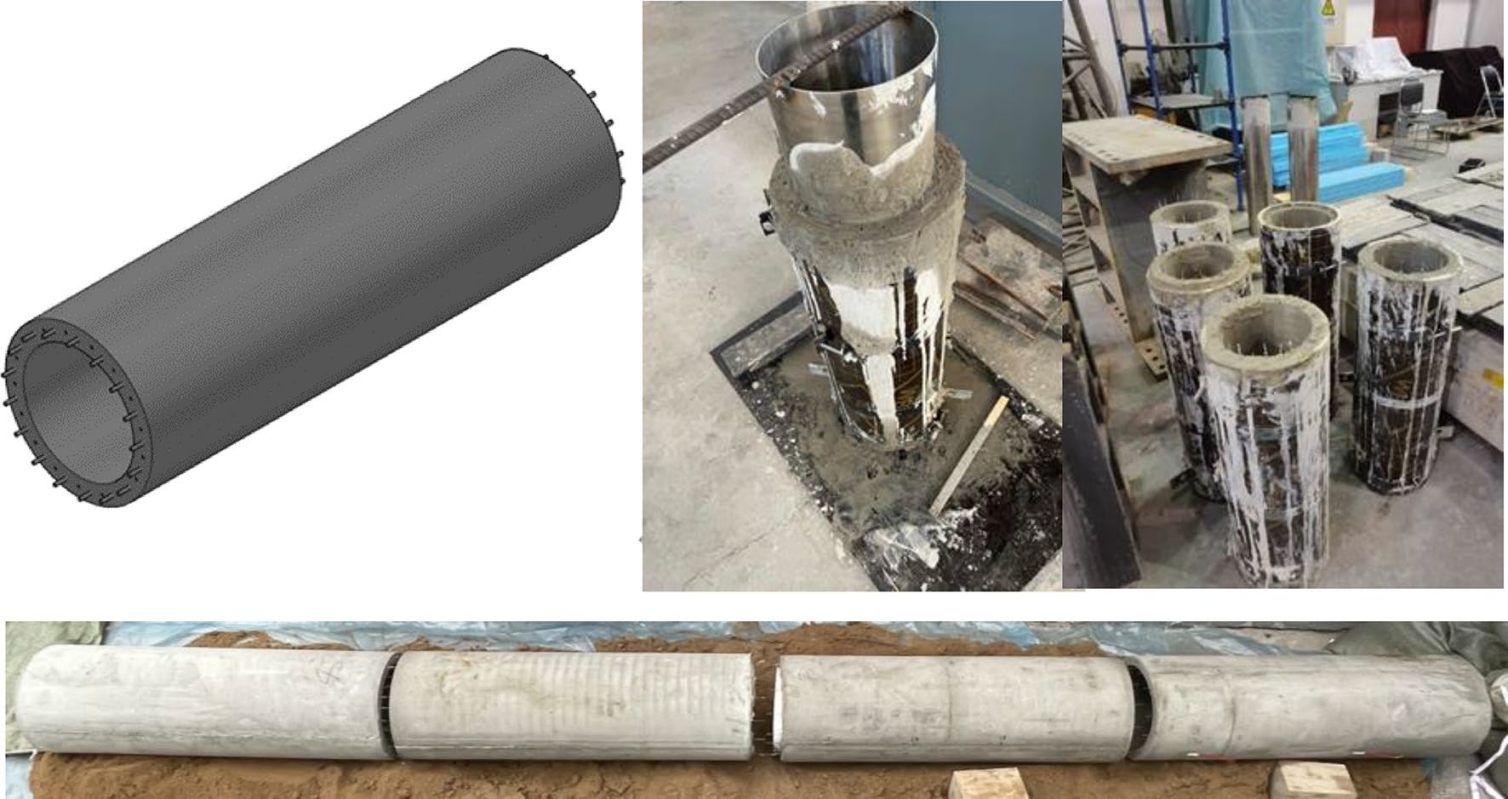
Tunnel model making diagram.

The final selection of the cement: gypsum: fine sand: water ratio is 5:5:30:11. The similarity ratio between the reinforcing mesh used in the test and the actual reinforcement in the project is 1:30, and the reinforcing mesh used is a 25 × 25 mm galvanized iron wire mesh with a diameter of 1 mm. The length of each section of the model is 800 mm. Pour the tunnel model according to the specified ratios and ensure that it is adequately cured until it reaches its required strength (Fig. 5). Twist together the protruding main bars and fill them with silicone weather-resistant sealant until the desired strength is achieved.

### Sensor deployment programme

**Figure 6:**
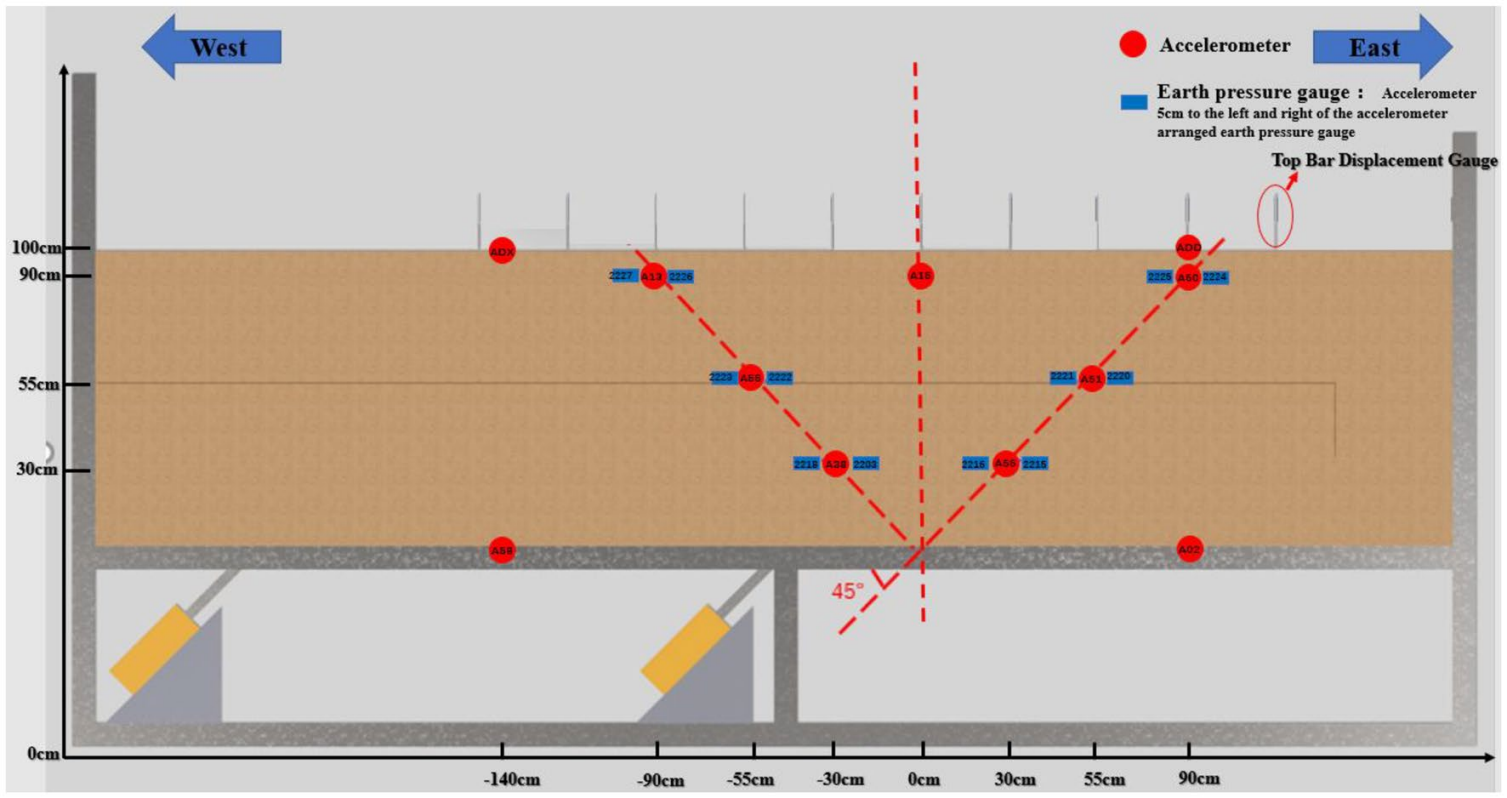
Schematic diagram for sensor placement in a sandy (clayey) soil site.

**Figure 7:**
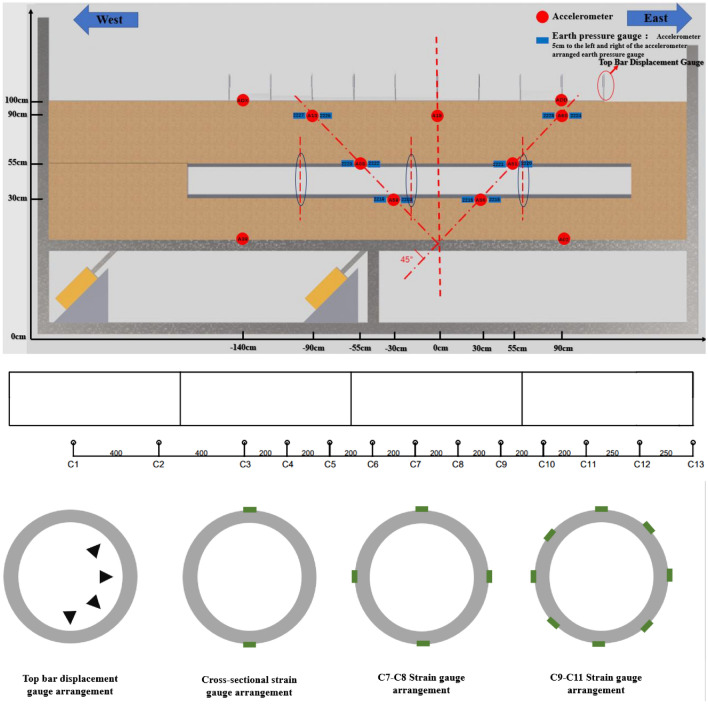
Schematic diagram for sensor placement in a site with a tunnel.

**Figure 8:**
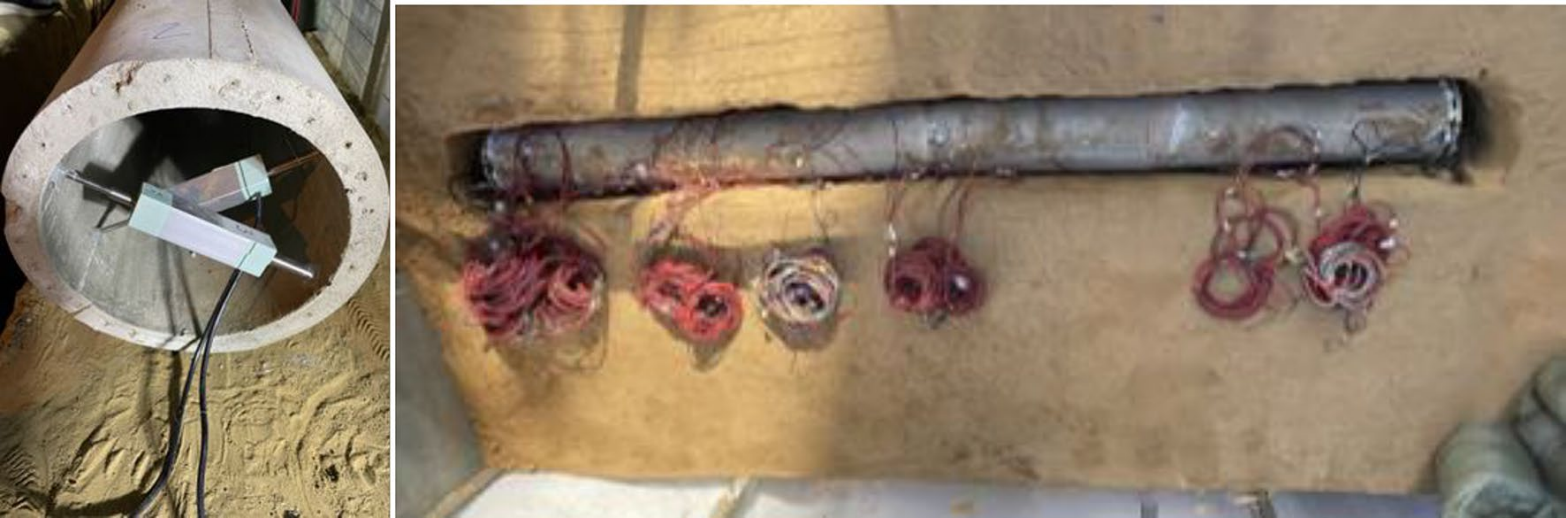
Schematic diagram illustrating the on-site placement of sensors.

The test data acquisition system is based on the system provided by Jiangsu Dong Hua Company. The primary sensors used in the system include the top-bar displacement gauge, earth pressure gauge, and accelerometer. Accelerometers and earth pressure gauges were installed at 30 cm, 55 cm, 90 cm, 100 cm, and the bottom plate of the soil body. Additionally, 11 top bar displacement gauges were positioned on the soil surface. The model strain gauges are primarily arranged in sections C1 to C13. Among them, 8 strain gauges are arranged in sections C9 to C11, 4 strain gauges are placed in sections C7 and C8, and the remaining sections have 2 strain gauges each, positioned at the model's arch top and arch waist. Based on the prediction that soil rupture is concentrated in the V-shaped rupture zone, four top-bar displacement gauges were arranged inside the segmental model interface on both sides. The specific arrangement of the sensor locations can be determined through a combination of pre-tests and analysis conducted by the subject group (Fig. 6, 7, 8).

## Comparative Analysis of Cross-Fault Tunnel Site Tests

### Comparative analysis of sites with a 45° inclination clay overburden on reverse faults and sites containing tunnels on them

**Figure 9:**
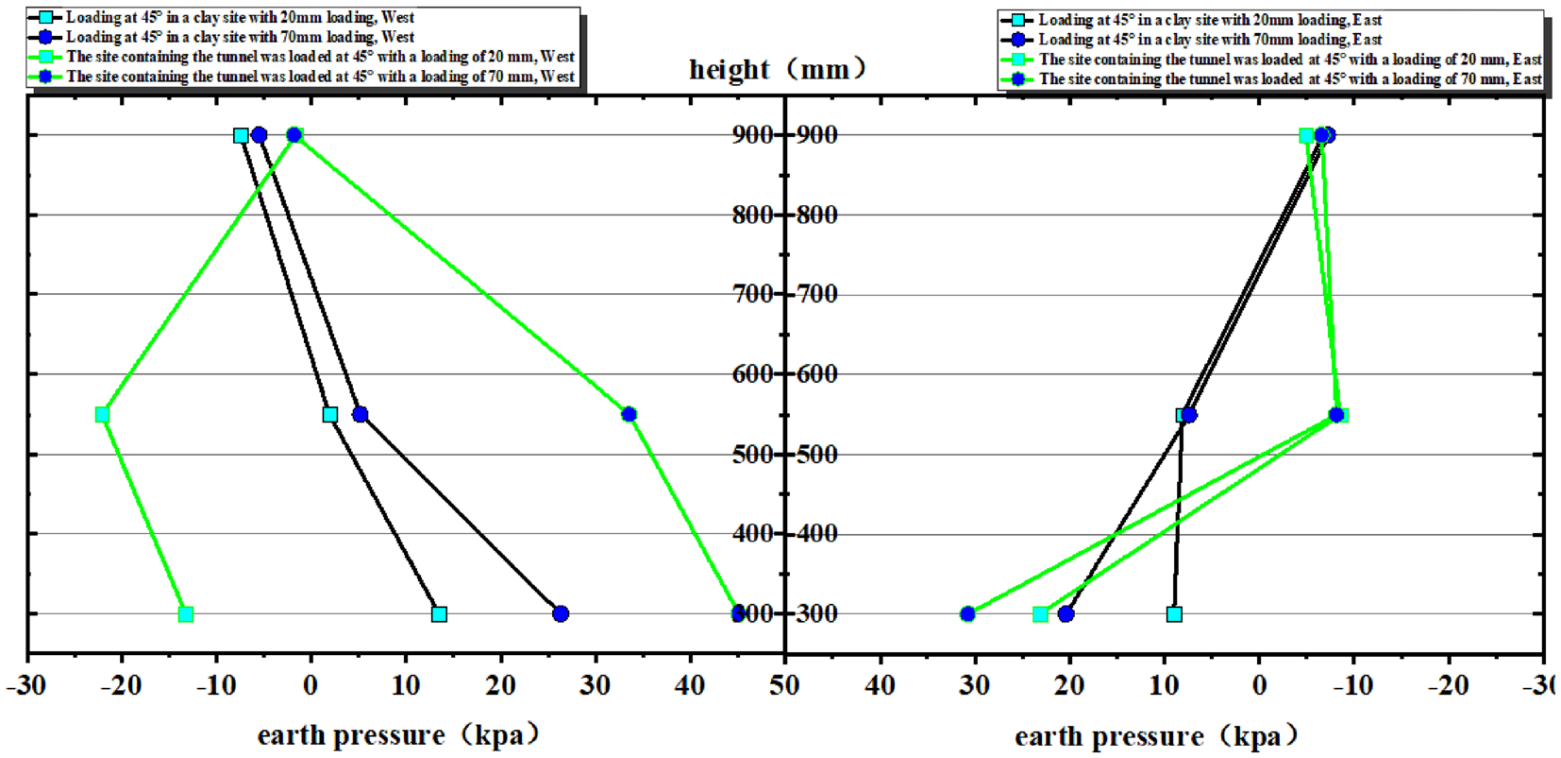
Comparison of earth pressure on the west side with that on the east side.

**Figure 10:**
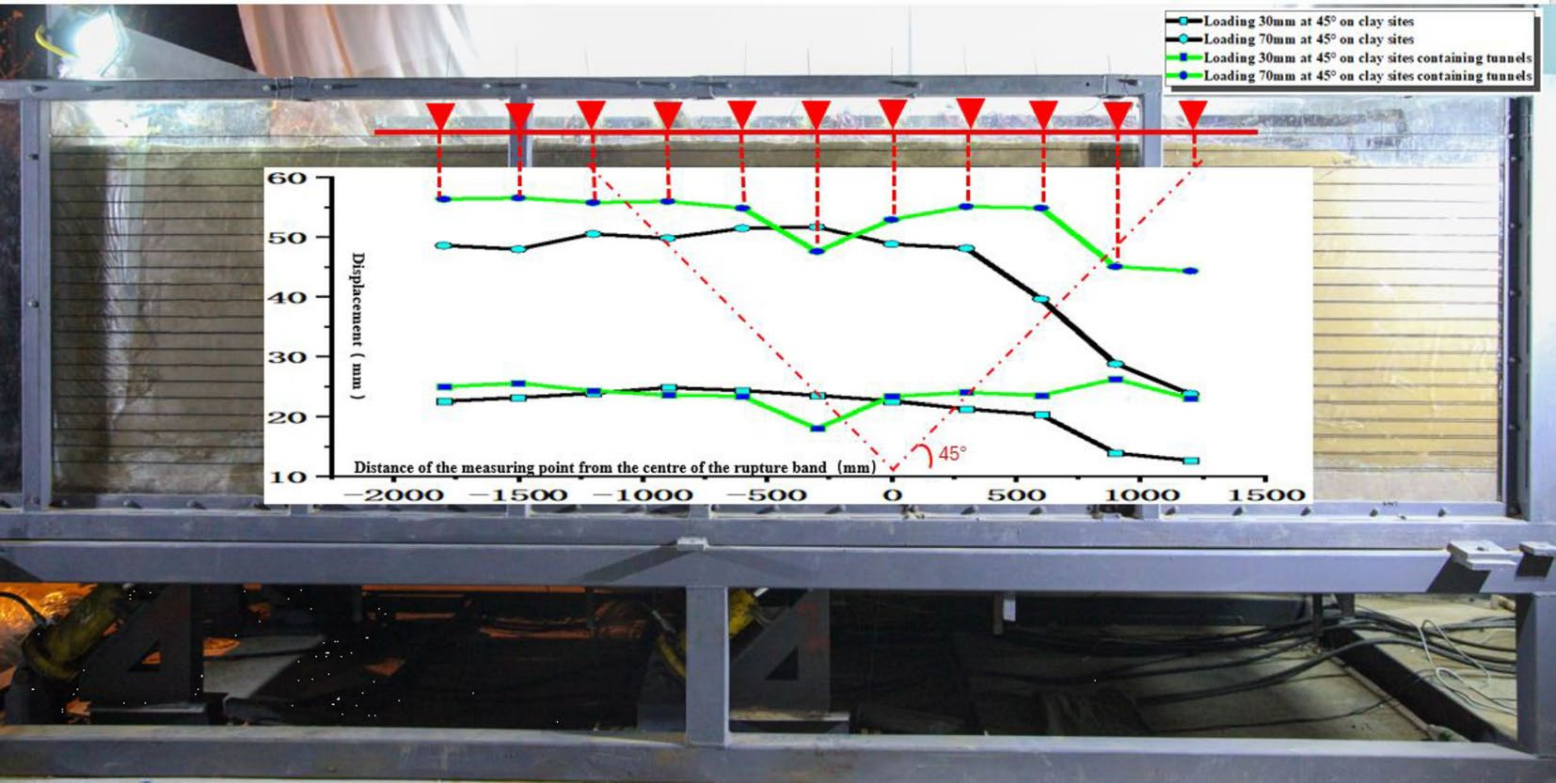
Comparison of the uneven deformation of the soil and the corresponding position of the top bar displacement gauge on the soil surface.

**Figure 11:**
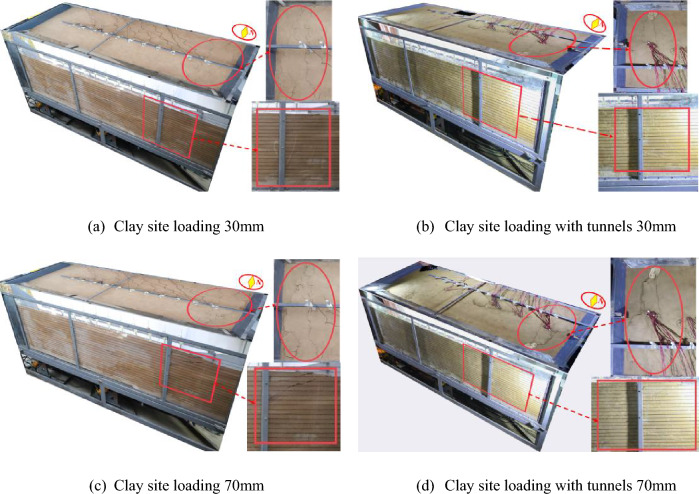
On-site test three-dimensional comparison diagram.

The clay site and its corresponding tunnel site tests, i.e., test numbers ① and ③.These included high-precision top-mounted displacement measurements for surface deformation, internal soil pressure monitoring using miniature pressure gauges, testing of active and passive discs for rock foundations, large dynamic acceleration measurement of the box-soil mass, and the tunnel model's strain test employing a small grid large range sensor. To this end, a systematic analysis of monitoring data was carried out. The specific analysis is as follows (Figs. 9 and 10):

The typical loading levels of 30 mm and 70 mm are chosen for analysis by comparing the front views and top views of the free clay site test ① and the containing tunnel site test ③. Based on the rupture traces, it is observed that the soil cracks in Test ① are more numerous and wider. When the loading amount reaches 30 mm, the soil on the east side of the front view bulges, and cracks appear on the surface of the soil on the west side. Additionally, as the loading amount increases, a crack emerges on the right side of the original crack at approximately 100 mm in the top view (Fig. 11a). During Test ③, when loaded to 30 mm, significant cracks appeared above the soil body on the west side in the front view. Additionally, two noticeable cracks appeared on the west side of the box in the top view, and there was substantial soil deformation (Fig. 11b).

When loaded to 70 mm, two cracks emerged in the soil above the east side of both Test ① and Test ③. The cracks in Test ① were wider, while those in Test ③ were longer and developed at a larger angle. On closer observation from the top view, it was noticed that both Test ① and Test ③ exhibited two rupture zones. The widths of the rupture zones were similar, but in Test ①, there were more, wider, and more prominent tiny cracks on both sides of the main rupture zone. Furthermore, the uplifted area of the rupture zone in Test ③ was higher. In Test ①, cracks started to appear on the ground surface at a loading amount of 10 mm. However, in Test ③, cracks appeared on the ground surface only when the loading amount reached 20 mm. As a result, when compared to tests ① and ③, test ① damage earlier (Fig. 11c,d).

In order to conduct a comparative analysis of the soil pressure on the east and west sides of each layer in Test ① and Test ③ (Fig. 9), it is observed that in Test ①, the soil pressure increases with the loading amount. Notably, at a depth of 300 mm, there is a significant change in the soil pressure. In Test ③, the soil pressure undergoes changes at depths of 300 mm and 550 mm, with the largest change occurring at 550 mm. In Test ①, significant changes in earth pressure are observed in the range of -300 ~ 300 mm from the center of the rupture zone at the position of 300 mm from the bedrock. However, the earth pressure undergoes little change at other positions. In Test ③, at a distance of 550 mm and 300 mm from the bedrock on the west side, the earth pressure exhibits significant changes with the increase in loading. On the east side, the earth pressure changes are not as pronounced with increasing loading. However, at a loading amount of 30 mm and a distance of 300 mm from the bedrock, both the 300 mm and 550 mm positions show higher values of earth pressure. Based on the analysis, it can be concluded that in Experiment ③, the tunnel structure is indeed under a significant threat at depths of 550 mm and 300 mm from the bedrock. Specifically, within the range of -550 mm to 550 mm and -300 mm to 300 mm from the center of the rupture zone, there is a serious risk to the integrity and stability of the tunnel structure. Based on the comparison between Test ① and Test ③, it is observed that the change in soil pressure at the center in Test ③ is greater than that in Test ①. This suggests that the area surrounding the tunnel structure, especially at the bedrock dislocation, needs to be strengthened in order to enhance the stability of the tunnel. Reinforcing the soil (surrounding rock) around the tunnel structure would be necessary in this case to ensure the safety and integrity of the tunnel under the increased soil pressure.

Based on the comparative analysis of high-precision top bar displacement and surface deformation, the displacement difference between Test ① and Test ③ at a loading amount of 30 mm does not show significant changes. However, as the loading amount increases to 70 mm, the soil body in Test ③ exhibits a larger lifting height compared to Test ①. This shows that the overall uneven deformation in test ③ is relatively large (Fig. 10, Table 3).

**Table 3:** Comparison and analysis table of uneven deformation in experiments.

	Range (mm)	Slope	Inclination angle (°)
Experiment ① Slope and inclination angle of inhomogeneous deformation	− 300 ~ 300	0.006	0.337
	300 ~ 900	0.034	1.922
	900 ~ 1200	0.022	1.241
Experiment ③ Slope and inclination angle of inhomogeneous deformation	− 600 ~ -300	0.024	1.38
	− 300 ~ 0	0.018	1.015
	600 ~ 900	0.033	1.88

**Figure 12:**
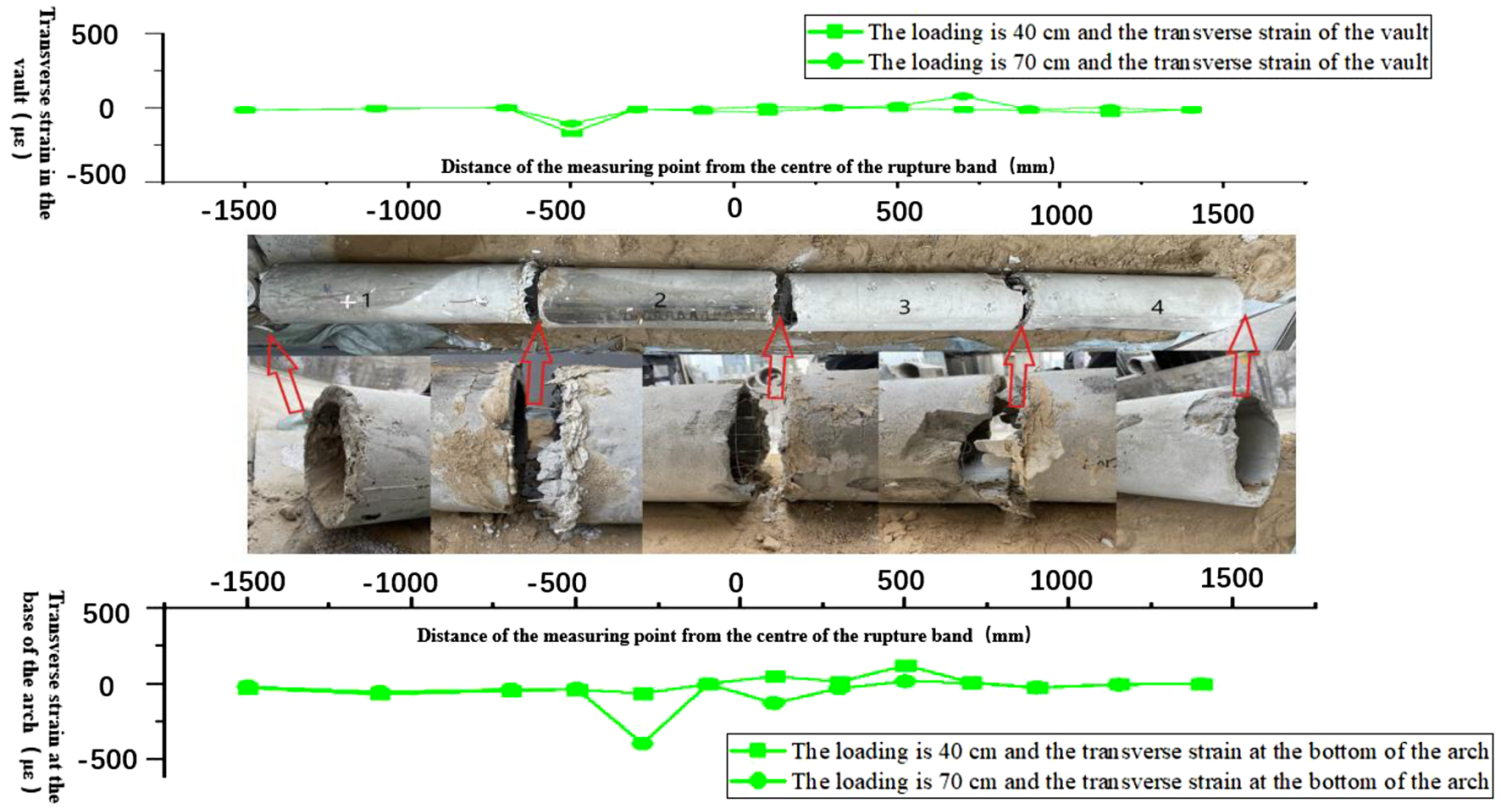
Comparison of overall structural damage to the tunnel.

According to the test ③ comparison diagram of the overall structural damage of the tunnel, the tunnel structure 2/3 and 3/4 pipe interface damage is serious. The western tunnel has minimal damage, while the eastern tunnel is severely damaged. The range of inhomogeneous deformation in test ③ is larger than that in test ①, and the rupture zone region is wider, so the range of soils for the rupture-resistant design of shallow tunnel structures is larger than their free fields. At the same time, this can be analyzed in the structural damage map of the tunnel in Test ③ (Fig. 12).

### Comparative analysis of the reverse fault 45° dip sand overburden site and its tunnel-bearing site

The sandy soil site and its corresponding tunnel site tests, i.e., test numbers ② and ④. These included high-precision top-mounted displacement measurements for surface deformation, internal soil pressure monitoring using miniature pressure gauges, testing of active and passive discs for rock foundations, large dynamic acceleration measurement of the box-soil mass, and the tunnel model's strain test employing a small grid large range sensor. To this end, a systematic analysis of monitoring data was carried out. The specific analysis is as follows:

Compared with test ②, the rupture zone area of test ④ obviously moved to the east. The scope of the soil uplift containing the tunnel site increased, the rupture zone migrated to the east side, the height of the soil uplift was higher, and the inhomogeneous deformation was obvious, which had the effect of "adding seismic" to the tunnel structure (Fig. 18).

Test ② When the loading amount was 30 mm, the soil body was lifted about 30 mm, but there was no soil rupture in the top view (Fig. 18a); when the loading amount of 70 mm, the sand soil was on the east side of the soil above a transverse crack. The top view found that the rupture was mainly concentrated on the east side, with the emergence of horizontal and vertical cracks and the soil bulging obvious (Fig. 18c). Test ④ When the loading amount was 30 mm the obvious soil bulged, top view found from the east side of the box 800 mm from the development of fine cracks to the middle position but did not penetrate the soil; near the edge of the box body, there is a crack that develops obliquely to the east side (Fig. 18b); loading to 70 mm did not appear obvious cracks; top view found that there are two cracks, the east side of the top of the crack is close to the edge of the box, and the east side of the bottom of the cracks away from the edge of the box of about 800 mm (Fig. 18d). Cracks appeared on the surface of test ② at a loading amount of 40 mm, and cracks appeared on the surface of test ④ at a loading amount of 30 mm. The surface of test ② was damaged before that of test ④.

**Figure 13:**
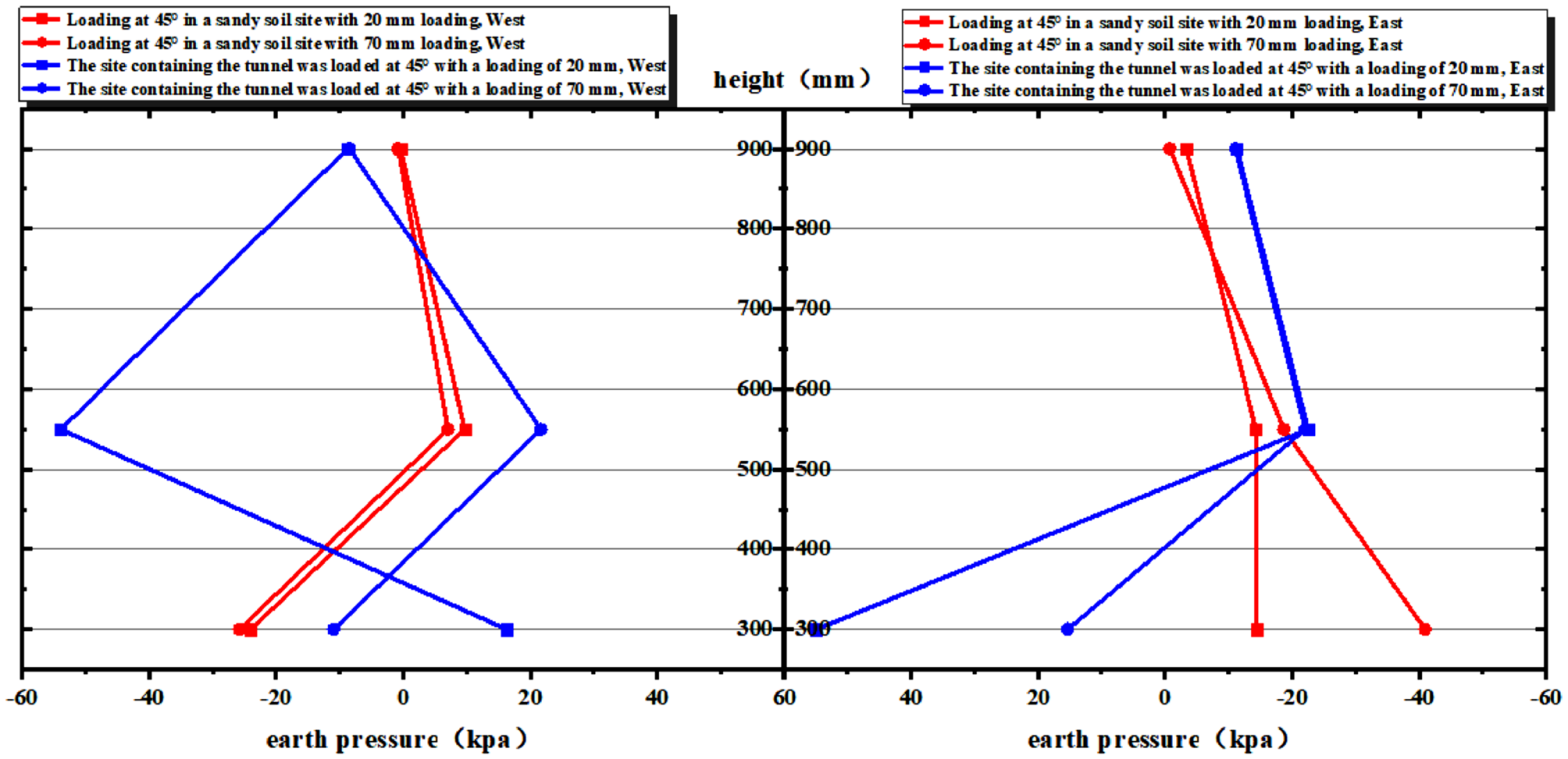
Comparison of earth pressure on the west side with that on the east side.

Comparative analysis of soil pressure in tests ② and ④(Fig. 13) shows that the soil pressure in test ② mainly changes in the middle of the soil body, with the most obvious change in the position of 300 mm and not much change in the other positions; test ④, after being put into the tunnel, shows that the change of soil pressure in the middle of the soil body is more significant; the variation of soil pressure on the west side of test ④ is greater than that on the east side; and the change in the position of 550 mm is more than that in the position of 300 mm, and the change in the position of the top of 900 mm basically does not have any change.

In Test ②, the change in earth pressure is significant within the range of − 300 mm–300 mm from the center of the rupture zone at the 300 mm position (Fig. 13).

In Test ④, the change in earth pressure is again evident within the range of − 300 mm–300 mm from the center of the rupture zone at the 300 mm position. Additionally, there is a noticeable change in earth pressure within the range of − 550 mm–550 mm from the center of the rupture zone at the 550 mm position. Thus, both of these ranges suggest potential serious damage to the tunnel structure (Fig. 13). Comparing the two tests, Test ④ demonstrates a greater change in earth pressure in the middle of the soil body compared to Test ②.

**Figure 14:**
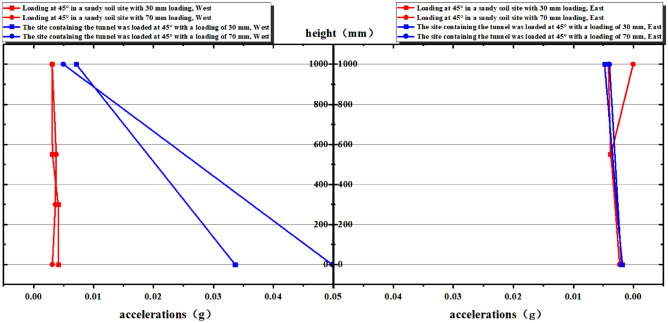
Comparison of peak acceleration on the east and west sides of the plot.

Based on the analysis of the valid data measured by accelerometers, which were placed along the east and west sides of the boundary of the "V" range of influence of 300 mm, 550 mm, and 900 mm, it was found that there was no significant change in acceleration on the east and west sides of tests ② and ④. Consequently, the peak acceleration in the middle of the soil layer remained relatively unchanged in both tests ② and ④ (Fig. 14).

**Figure 15:**
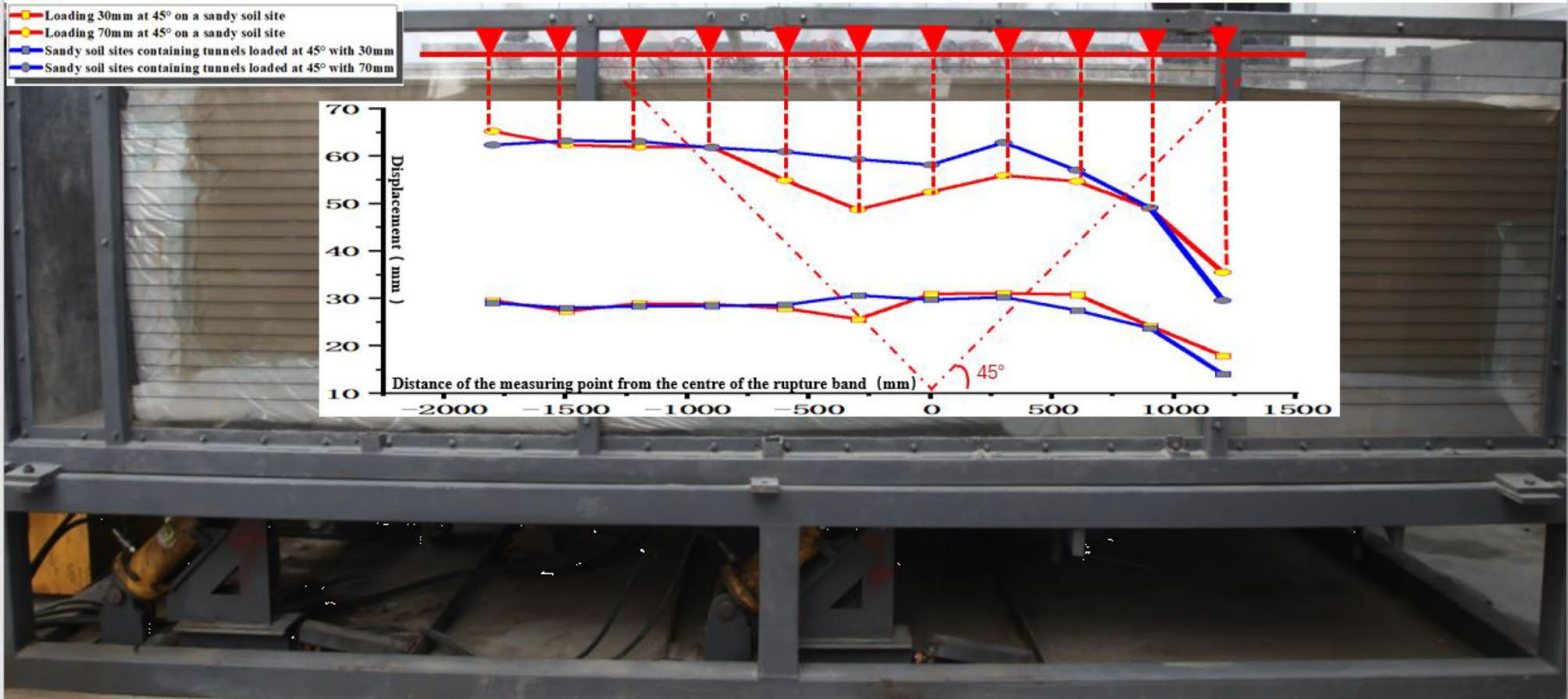
Comparison of the uneven deformation of the soil and the corresponding position of the top bar displacement gauge on the soil surface.

The reaction spectrum is further analyzed. The results show that the platform value of the west side of the soil surface in both Test ② and Test ④ is larger, with a value of 2.0. The platform value of the bedrock active disk is 1.5 (Fig. 15).

**Figure 16:**
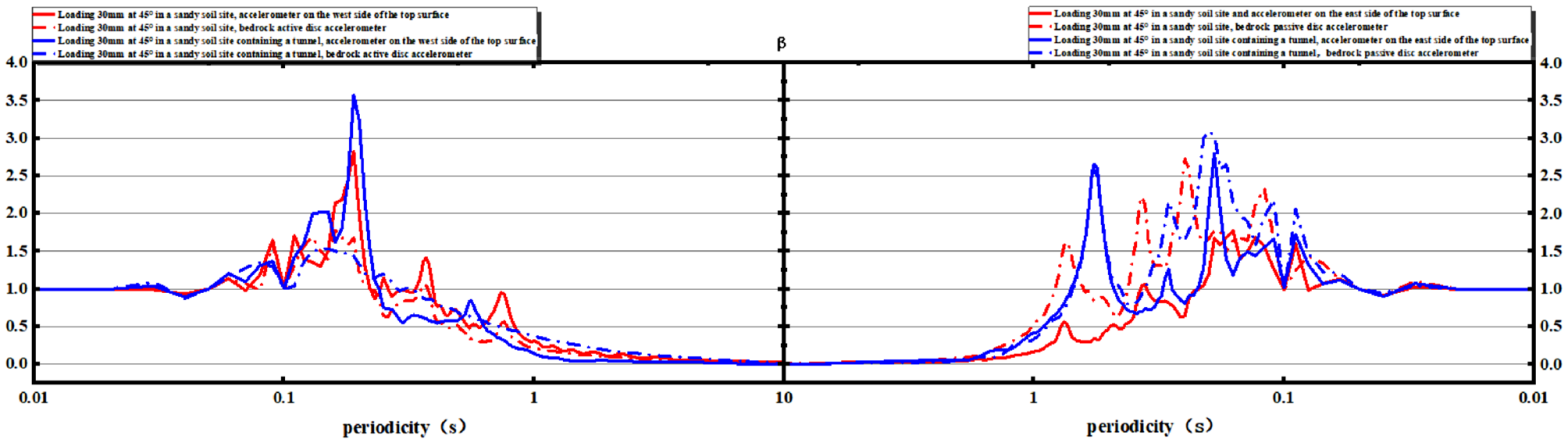
Comparison of the reaction spectra of the east side and west side with a loading volume of 30 mm.

**Figure 17:**
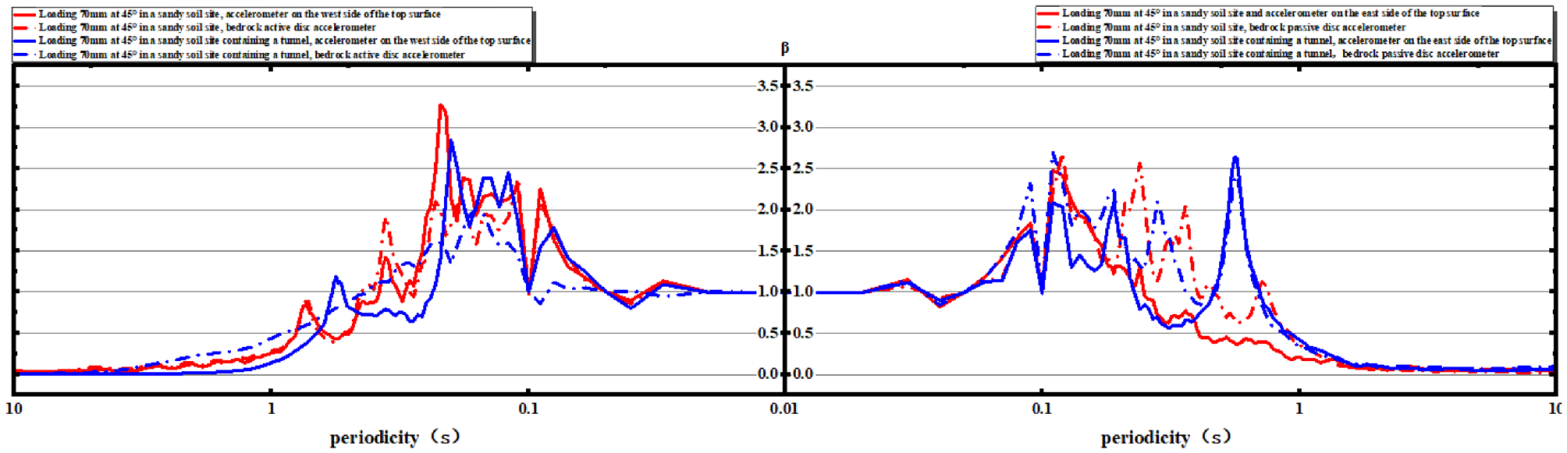
Comparison of the reaction spectra of the east side and west side with a loading volume of 70 mm.

With an increase in the loading amount, the platform value of the west side of the bedrock active disk increases to 2.5 when the loading reaches 70 mm. However, there is no significant change in the platform value for the east side of the soil surface and the bedrock active disk as the loading amount increases (Figs. 16, 17).

**Figure 18:**
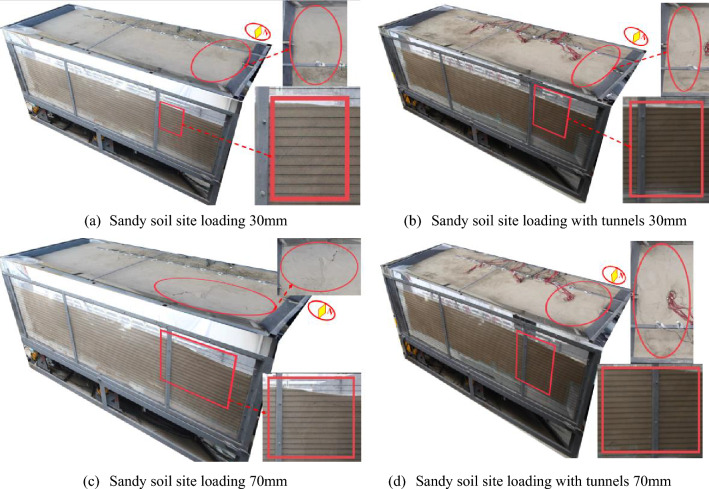
On-site test three-dimensional comparison diagram.

Therefore, it can be concluded that in the influence zone of the rupture, the tunnel outside this zone can be designed using the reaction spectrum theory in the conventional seismic design method, as there are no significant changes observed in the platform values for the east side of the soil surface and the bedrock active disk with the increase in the loading amount (Fig. 18).

In Test ② and Test ④, the top bar displacement is indeed approximately the same when the loading amount is 30 mm. However, as the loading amount increases, Test ② shows an increased deformation difference in the rupture zone from the center position, ranging from -900 mm to 600 mm. Furthermore, in the region of 600 mm to 1200 mm, both tests exhibit significant and uneven deformation (Fig. 15, Table 4).

**Table 4:** Comparison and analysis table of uneven deformation in experiments.

	Range (mm)	Slope	Inclination angle (°)
Experiment ② Slope and inclination angle of inhomogeneous deformation	− 900 ~ -300	0.027	1.535
	− 300 ~ 600	0.008	0.460
	600 ~ 1200	0.035	1.981
Experiment ④ Slope and inclination angle of inhomogeneous deformation	0 ~ 300	0.016	0.898
	300 ~ 900	0.023	1.311
	900 ~ 1200	0.065	3.730

**Figure 19:**
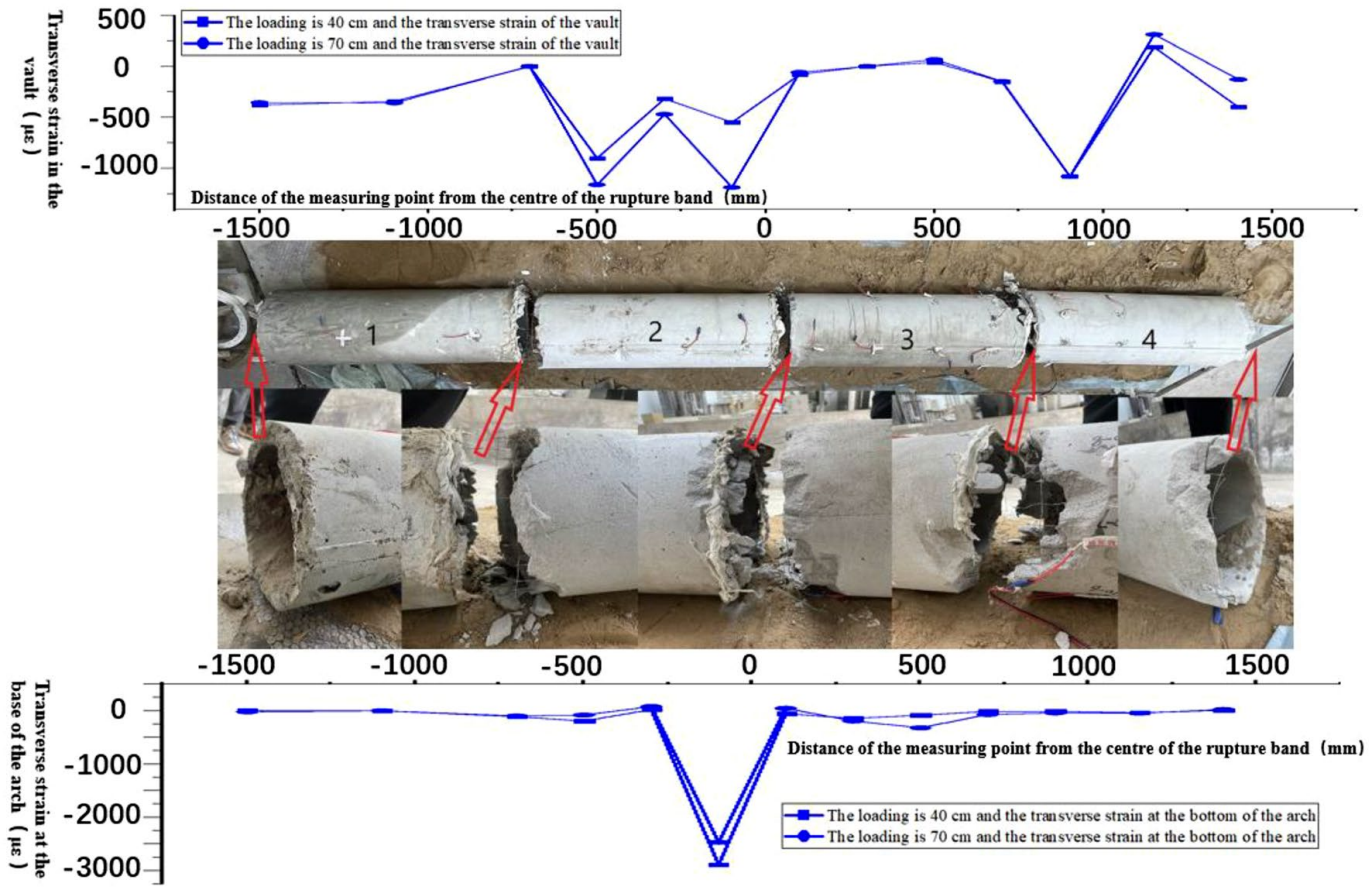
Comparison of overall structural damage to the tunnel.

Based on the comparative diagram of the overall structural damage of the tunnel in Test 4, it is evident that severe damage occurred at the interface of tubes 2/3 and 3/4 of the tunnel structure. Damage to the western tunnels is less severe, while damage to the eastern tunnels remains severe. The range of inhomogeneous deformation in Test ④ is larger than that in Test ②, and the rupture zone region is wider, so the range of soils designed for rupture resistance of shallow tunnel structures is larger than their free fields. Also, this can be analyzed in the damage map of the tunnel structure in test ④ (Fig. 19).

The comparison of tests ③ and ④ (Figs. 12 and 19), analyzed under the same inclination angle at the sandy soil site and the clay site, reveals a larger rupture range in the sandy soil site tunnel structure. At the tunnel interface, they are all severely damaged. Therefore, when the reverse fault is the same as the fault dip, the sandy soil site is more dangerous to the underground tunnel structure, and both sites need to strengthen the seismic design of the main tunnel structure and joints.

## Conclusion

This article adopts the design concept of "fuse", which is to use segmented flexible joint tunnel segments during fault displacement, based on the principle of "sacrificing small for large", to reduce the overall damage of soil and surrounding rock to the length of the tunnel body. It’s analyzed that the failure mechanism of segmented tunnel structures in sandy and cohesive soil sites during low-dip reverse fault displacement.

1. The tunnel structure's failure characteristics in the soil field across the fault cover layer are determined not only by the degree of bedrock dislocation but also by the failure range of soil with different properties under the action of dislocation. As a result, the position and shape of the overburden soil rupture range induced by bedrock dislocation at different angles should be obvious in the design, particularly the longitudinal soil rupture range in the buried depth where the tunnel is placed.
2. Compared with the free field of cohesive soil overburden, the uneven deformation of the surface of the cohesive soil site with tunnel structure becomes slower as a whole. With the increase in bedrock dislocation, the downward deformation at the tunnel structure at the reverse position of the bedrock dislocation dip angle is significant. The variation of internal earth pressure in the hanging wall area is about 2–4 times that of its free field. In the clay, the damage to the arch bottom of the tunnel structure is more serious, and the strain change is about twice that of the vault. From the longitudinal length of the tunnel, the strain range at the bottom of the arch is about 2 times the length of the top of the arch.
3. Compared with the free field of sand overburden, the surface uneven deformation of the sand site with tunnel structure does not slow down as a whole. With the increase in bedrock dislocation, the downward deformation of the tunnel structure in the V-shaped extrusion shear rupture zone composed of bedrock dislocation dip angle and its reverse position is significant. The length of the influence range is about 5 times that of the free field, and the maximum inclination angle is about 1.8 times that of the free field. The internal earth pressure change in the hanging wall area is about 3–5 times that of the free field. For the ground main rupture zone region across the tunnel length direction, the peak acceleration of the tunnel structure is significantly changed to about 3–5 times of the free field, and the long-period effect of the response spectrum in this region is obvious, and the characteristic period can reach 1 s faster. In sandy soil, the damage to the arch bottom of the tunnel structure is more serious, and the strain change is about three times that of the vault. However, from the longitudinal length of the tunnel, the strain variation range at the top of the arch is about 2 times the length of the bottom of the arch.
4. Compared with the tunnel structure site of cohesive soil, the strain change of the tunnel structure in the sandy soil site is more significant, and the arch top is about 2 times and the arch bottom is about 6 times. In addition, the uplift range of the sand cover layer increases, the rupture zone migrates to the right, the lifting height of the soil is higher, and the uneven deformation is obvious. Therefore, the sandy soil site plays an " adding seismic " role in the cross-fault tunnel structure.
5. In the area spanning the ground fault rupture zone, the tunnel structure is affected by soil compression and shear failure. The main structure of the tunnel and the pipeline interface are vulnerable to serious threats. Therefore, it is necessary to take anti-rupture measures, such as over-excavation design, segmented flexible connection, damping joint, damping layer, etc^17–20^.
6. This paper utilizes a substantial tunnel model test apparatus to carry out field testing on various soil cover layers over low-dip reverse faults. The experimental data offer valuable insights for the structural design and construction of tunnels across fault rupture.

## Data Availability

The datasets used and/or analyzed during the current study available from the corresponding author on reasonable request.
